# Keratoconus: cross-linking the window of the eye

**DOI:** 10.1177/26330040211003573

**Published:** 2021-03-31

**Authors:** Sally Hayes, Siân R. Morgan, Keith M. Meek

**Affiliations:** Structural Biophysics Research Group, School of Optometry and Vision Sciences, Cardiff University, Maindy Road, Cardiff, CF24 4HQ. UK; Structural Biophysics Research Group, School of Optometry and Vision Sciences, Cardiff University, Cardiff, UK; Structural Biophysics Research Group, School of Optometry and Vision Sciences, Cardiff University, Cardiff, UK

**Keywords:** collagen, cornea, cross-linking, keratoconus, riboflavin, UVA

## Abstract

Keratoconus is a condition in which the cornea progressively thins and weakens, leading to severe, irregular astigmatism and a significant reduction in quality of life. Although the precise cause of keratoconus is still not known, biochemical and structural studies indicate that overactive enzymes within the cornea break down the constituent proteins (collagen and proteoglycans) and cause the tissue to weaken. As the disease develops, collagen fibres slip past each other and are redistributed across the cornea, causing it to change shape. In recent years, it was discovered that the photochemical induction of cross-links within the corneal extracellular matrix, through the use of riboflavin and ultraviolet (UVA) light, could increase the strength and enzymatic resistance of the tissue and thereby halt keratoconus progression. Worldwide acceptance and use of riboflavin/UVA corneal cross-linking therapy for halting keratoconus progression has increased rapidly, in accordance with the growing body of evidence supporting its long-term effectiveness. This review focusses on the inception of riboflavin/UVA corneal cross-linking therapy for keratoconus, its clinical effectiveness and the latest scientific advances aimed at reducing patient treatment time, improving patient comfort and increasing patient eligibility for treatment.

**Plain language summary:**

**Review of current treatments using cross-linking to halt the progress of keratoconus**

Keratoconus is a disease in which the curved cornea, the transparent window at the front of the eye, weakens, bulges forward into a cone-shape and becomes thinner. This change of curvature means that light is not focussed onto the retina correctly and vision is progressively impaired. Traditionally, the effects of early keratoconus were alleviated by using glasses, specialist contact lenses, rings inserted into the cornea and in severe cases, by performing a corneal transplant. However, it was discovered that by inducing chemical bonds called cross-links within the cornea, the tissue could be strengthened and further thinning and shape changes prevented. The standard cross-linking procedure takes over an hour to perform and involves the removal of the cells at the front of the cornea, followed by the application of Vitamin B2 eye drops and low energy ultraviolet light (UVA) to create new cross-links within the tissue. Clinical trials have shown this standard procedure to be safe and effective at halting keratoconus progression. However, there are many treatment modifications currently under investigation that aim to reduce patient treatment time and increase comfort, such as accelerated cross-linking procedures and protocols that do not require removal of the surface cells. This review describes the different techniques being developed to carry out corneal cross-linking efficiently and painlessly, to halt keratoconus progression and avoid the need for expensive surgery.

## Background

Keratoconus is a condition in which the cornea becomes progressively thinner and weaker over time, leading to an outward bulging of the tissue and severe, irregular astigmatism. Although keratoconus is a relatively rare disease that does not normally result in blindness, it does have a significant impact on public health due to its early age of onset and the severely detrimental impact that it has on patient quality of life.^1^ The condition typically manifests in young adults in their teens to early twenties and in some, but not all, cases it may progress for up to 10–20 years thereafter, with the rate of progression commonly being greater in paediatric patients (<18 years of age) than in other age groups.^2^ Estimates of keratoconus prevalence vary from 17 to 4000 per 100,000 of the general population, with the large variation being attributed to differences in geographical location, diagnostic methods, sample size and study design.^3^

Although the precise cause of keratoconus and the mechanism by which it progresses remain a matter of uncertainty, a number of environmental and genetic risk factors have been identified, such as excessive eye rubbing, atopy and a family history of keratoconus.^3,4^ In addition to these risk factors, the keratoconus cornea also exhibits a number of biochemical and structural abnormalities that likely contribute to disease progression. For example, collagen degradation is enhanced in the keratoconic cornea by the presence of higher than normal levels of proteolytic enzymes and reduced levels of proteinase inhibitors.^5,6^ Additionally, X-ray scattering studies of advanced stage keratoconus corneas have revealed abnormalities in the arrangement and distribution of the stromal collagen lamellae that form the bulk of the cornea and are believed to help the healthy cornea withstand the forces acting upon it to maintain its curvature.^7–11^ Such findings indicate that inter-fibrillar and inter-lamellar slippage, and a redistribution of collagen mass are involved in the progressive thinning and steepening in keratoconus corneas,^8^ a process which would undoubtedly be facilitated by the enhanced degradation of collagen in these corneas, as well as by the reduced lamellar interweaving and fewer lamellar insertions into Bowman’s membrane that are associated with the condition.^12,13^

Visual function may be improved through the use of glasses or specialist contact lenses,^14^ or through the insertion of intra-corneal ring segments (Intacs or Ferrara Rings)^15^ or a flexible, full ring intra-corneal implant (MyoRing),^16^ which flattens the curvature of the keratoconus cornea and, in the case of the MyoRing, may also strengthen and stabilise the weakened tissue.^17^ However, these management tools alone are unable to address both the enhanced enzymatic digestion and tissue weakening associated with keratoconus to stop the natural progression of the condition. In approximately 12% of cases, invasive surgical treatment in the form of a full or partial thickness corneal transplant is required due to severe disease advancement, contact lens intolerance and/or corneal scarring.^18^ In 2012, a survey of 184,576 corneal transplants performed in 116 countries revealed keratoconus to be one of the leading indications for corneal transplantation (accounting for 27% of all transplants).^19^ Whilst corneal transplantation has a notably high success rate (with graft survival rates of >90% at 13 years),^20^ the procedure requires a protracted recovery time, the use of corticosteroids to prevent rejection and the continued use of rigid contact lenses to restore visual function. Corneal transplantation also carries the risk of serious complications such as infection, graft failure (and in rare cases recurrent keratoconus) and, as a treatment option, it is susceptible to worldwide shortages of donor corneas.^19,21,22^ Although transplantation remains the dominant, end of line, surgical intervention for advanced keratoconus, management of the condition has changed dramatically since the early 2000s with the introduction of photochemical corneal cross-linking therapy – a minimally invasive treatment proven to halt early-stage progressive keratoconus. This review will focus on the development and recent advances in corneal cross-linking therapy for keratoconus.

## Inception of corneal cross-linking therapy for keratoconus

Since keratoconus progression is rarely seen in patients >40 years old due to the natural stiffening of the cornea with age (as glycation-induced cross-links become more prevalent within the stromal extracellular matrix),^23,24^ it was postulated that artificially cross-linking the extracellular matrix of the keratoconus corneal stroma might provide a protective effect against enzymatic degradation and fibrillar slippage.^25^ Spoerl *et al.* went on to show that application of a photosensitiser (riboflavin) in conjunction with a 30-min exposure to 370 nm ultraviolet (UVA; corresponding to one of the absorption maxima of riboflavin), resulted in significant corneal stiffening and an increased resistance of the tissue to enzymatic digestion.^25,26^

Riboflavin-5 phosphate (vitamin B2) was selected as the photosensitiser of choice on the basis of its water solubility and biocompatibility, and the fact that it readily diffuses into the corneal stroma when the epithelium is removed. Riboflavin plays a crucial role in the cross-linking process; it enhances cross-link formation and absorbs UVA as it passes through the corneal stroma, thereby protecting the deeper ocular structures, such as the lens and retina, from damage.^27^ Oxygen is also highly important to the process.^28^ When exposed to UVA light, the riboflavin is excited to singlet and triplet states, and follows one of two photochemical reactions. The Type I mechanism predominates at low oxygen concentrations and results in the generation of riboflavin free radical species, which interact with molecular oxygen in the ground state and form oxidation products. At higher oxygen concentrations (present at the start of the procedure), the Type II mechanism predominates, with riboflavin transferring energy to oxygen in the ground state to generate more reactive singlet molecular oxygen, which then reacts with stromal extracellular components to induce covalent cross-link formation.^29^ Extensive laboratory studies of the interactions between collagens and proteoglycans during cross-linking,^30,31^ coupled with investigations into the hydrodynamic behaviour and structure of cross-linked corneas, indicate that the cross-links are most likely formed within and between collagen molecules on the fibril surfaces, and within the proteoglycan core proteins that reside between the collagen fibrils.^32^

## Clinical effectiveness and limitations of the standard cross-linking protocol

**Figure 1.**
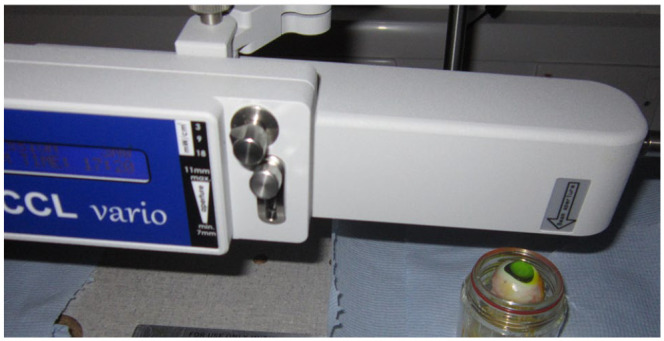
Laboratory set-up (authors own) for riboflavin/UVA corneal cross-linking of an enucleated porcine eye. Note the bright yellow fluorescence of the stromal riboflavin as the cornea is exposed to UVA light from above. UVA, ultraviolet.

The standard cross-linking procedure, often referred to as the ‘Dresden protocol’, is an ‘epithelium-off’ technique that requires a minimal corneal thickness of 400 µm to ensure sufficient shielding of the endothelium, lens and retina.^27^ At the start of the procedure, the central 8 mm of the corneal epithelium is fully removed to facilitate the penetration of riboflavin solution (comprising 0.1% riboflavin-5-phosphate and 20% dextran) into the stroma. During a 30-min instillation period, the riboflavin solution is re-applied at frequent intervals to ensure saturation of the stroma. The cornea is then irradiated with 3 mW/cm^2^ of UVA for 30-min (total radiance of 5.4 J/cm^2^), with continued re-application of riboflavin throughout (Figure 1).

Wollensak *et al.* first demonstrated the clinical potential of corneal cross-linking for the management of progressive keratoconus in a pilot study involving 23 cases of moderate to advanced keratoconus, in which a cessation of progression was reported in all cases.^33^ The safety and effectiveness of the procedure was subsequently supported by a number of other non-randomised studies (the largest of which involved 272 patients^34^), and randomised-controlled trials (RCTs; summarised in Table 1). The treatment has been shown to have no effect on endothelial cell density and morphology at 5-year follow up,^35^ and the pre-operative thickness and stratification of the corneal epithelium is restored within 3–6 months of treatment.^36^ Although cross-linking results in a loss of keratocytes from the anterior and mid-stroma (up to a depth of ~300 µm) within 24 h of treatment,^37^ the effect is temporary, with stromal keratocyte repopulation being initiated within 3-months and reaching completion within 6 months.^38^ At approximately 1 month post-treatment a demarcation line (an area of hyper-reflectivity) is visible, with anterior segment optical coherence tomography and confocal microscopy at a depth of ~300 μm. Some have suggested that this line may be a wound-healing effect, whilst others have purported that it may represent the border between treated and untreated tissue (and therefore be used as an indicator of the effective depth of cross-linking).^39^ However, in a recent study in which an ultra-high speed Scheimpflug camera coupled with a non-contact tonometer (Corvis ST) was used to measure *in vivo* dynamic corneal response parameters in 66 cross-linked keratoconus corneas, it was concluded that the precise location of the demarcation line did not appear to be related to corneal stiffening.^40^

**Table 1.** RCTs (>12 months) examining the efficacy of standard protocol riboflavin/UVA corneal cross-linking in halting keratoconus progression.

Source	Study parameters	Primary outcomes	Study limitations
Greenstein *et al.*^41^	71 eyes (49 with keratoconus; 22 with post-LASIK ectasia); 12-month follow up	CXL group: improvement in 4/7 topography indices indicating improved corneal shape. Untreated fellow eye and sham controls: no change in topography from baseline.	Relatively short follow-up; participants in the control group allowed to cross-over to the treatment group after 3-months; patients with keratoconus and ectasia treated as a single cohort
O’Brart *et al.*^42^	24 participants with early/moderate bilateral keratoconus; 18-month follow up	CXL group: ↓ corneal steepness; no evidence of keratoconus progression. Untreated fellow eyes: majority remained stable but evidence of keratoconus progression in 14% of eyes.	It was not possible to mask the participants due to the invasive nature of the treatment.
Wittig- Silva *et al.*^43^	100 eyes with progressive keratoconus; 36-month follow up	CXL group: overall ↓ corneal steepness (Kmax ↓ 1.03 D) and a cessation of keratoconus progression in all but one eye. Untreated controls: Kmax ↑ 1.75 D	After 6 months, control group participants with evidence of keratoconus progression were allowed to cross over into the treatment group leading to a possible underestimation of the treatment effect.
Lang *et al.*^44^	29 eyes with early progressive keratoconus; 36 months	CXL group: overall ↓ corneal steepness (Kmax ↓ 0.35 D/year) but in 4/15 patients there was a yearly ↑ Kmax of 0.02–0.32 D. Sham treated controls: evidence of corneal steepening in 8/14 patients	Relatively small sample size due to recruitment issues as participants became increasingly unwilling to accept sham procedures.
Seyedian *et al.*^45^	26 patients with bilateral keratoconus; 12-month follow up	CXL group: overall ↓ corneal steepness (Kmax ↓ 0.2 D); cessation of keratoconus progression in all but three eyes. Untreated fellow eye: Kmax ↑ 0.4 D	Relatively short follow up. It was not possible to mask the participants due to the invasive nature of the treatment.
Hersh *et al.*^46^	205 patients with progressive keratoconus; 12 months	CXL group: overall ↓ corneal steepness (Kmax ↓ 1.6 D); Kmax ↓ by 2.0 D or more in 28 eyes (31.4%) and ↑ by 2.0 D or more in 5 eyes (5.6%). Sham treated controls: continuation of keratoconus progression	Relatively short follow up. Participants in the control group were allowed to cross over to the treatment group at 3 months; only two control eyes remained at 12-months follow up.

BCVA, best corrected visual acuity; CXL, cross-linking; Kmax, measurement of treatment outcomes: maximum simulated keratometry; LASIK, laser-assisted in situ keratomileusis; RCT, randomised controlled trial; UVA, ultraviolet.

The long-term corneal stabilisation and sustained improvement in corrected distance visual acuity achieved with the standard cross-linking protocol has been evidenced in numerous clinical studies with follow-up times of up to 10 years.^34,47,48^ However, in a small minority of cases, uncontrolled and continued long-term flattening of corneal curvature has been reported.^49^ Despite the evident success of the ‘standard’ cross-linking protocol for halting keratoconus progression, it is not without limitations. For example, it requires a relatively long treatment time (~1 h), patients with corneas of <400 µm are not eligible for treatment, and the removal of the epithelium causes significant patient discomfort and carries a risk of infection.^50,51^ These factors have driven the development of accelerated and transepithelial cross-linking procedures that offer the advantage of increased patient comfort, easier treatment of children and less co-operative patients, and improved cost-effectiveness.

## Development of accelerated cross-linking protocols

Based on the Bunsen-Roscoe Law of reciprocity,^52^ it was proposed that the same biological effect as achieved with the standard (3 mW/cm^2^ for 30 min) protocol could be accomplished using a higher fluence for a shorter period of time, so long as the total energy dose of 5.4 J/cm^2^ was kept constant for example, 9 mW/cm^2^ UVA for 10 min, 18 mW/cm^2^ UVA for 5 min or 30 mW/cm^2^ UVA for 3 min. Laboratory investigations into the effectiveness of accelerated cross-linking (ACXL) protocols have produced somewhat conflicting results. For example, the stress–strain measurements of Schumacher *et al.* from strips of cross-linked porcine corneas showed that a 9-min exposure to 10 mW/cm^2^ UVA resulted in a similar increase in corneal stiffness as that achieved with the standard protocol.^53^ However, Hammer *et al.* showed that, although a 10-min exposure to 9 mW/cm^2^ UVA stiffened the porcine cornea, it was to a lesser extent than that achieved with the standard protocol, and the stiffness of corneas cross-linked with 18 mW/cm^2^ for 5 min did not differ from that of untreated corneas.^54^ *In vivo* measurements of human dynamic corneal response at 2-years post-cross-linking indicate that both the 9 mW/cm^2^ (10 min) and 18 mW/cm^2^ (5 min) procedures result in significant corneal stiffening,^55^ and *ex vivo* studies show increased enzymatic resistance following standard and ACXL protocols (up to 18 mW/cm^2^ for 5 min) with only subtle differences between treatments.^56^ However, the enzymatic resistance of corneas treated with the standard protocol was far greater than those treated with 30 mW/cm^2^ for 5 min, suggesting a reduced cross-linking effect and a failure of the Bunsen–Roscoe law of reciprocity at higher UVA intensities.^57^ Supporting this, biomechanical studies have also shown a sudden decrease in cross-linking efficacy when very high UVA intensities (greater than 45 mW/cm^2^) are used.^58^

RCTs have demonstrated the efficacy of ACXL (9 mW/cm^2^, 18 mW/cm^2^ and 30 mW/cm^2^) at halting keratoconus progression in the short term^59,60^ and at 4 years follow up, cross-linking with 18 mW/cm^2^ for 5 min was found to be similarly effective as the standard procedure.^61^ However, more RCTs are required to confirm the long-term efficacy of this and other ACXL procedures (especially those using very high UVA intensities).

## Pulsed and oxygen supplemented cross-linking protocols

It has been suggested that the apparent failure of the Bunsen-Roscoe Law of reciprocity at very high levels of UVA intensity may be due to the more rapid oxygen depletion that occurs when using such high intensity UVA as well as to the fixed rate of oxygen diffusion into the cornea, limiting the cross-linking process.^62^ This has led to a further wave of suggested ACXL treatment modifications, including the extension of the exposure time by 30–40% to increase the overall energy dose.^63,64^ The potential benefit of an extended ACXL procedure is supported by laboratory studies showing that increasing the exposure time from 3 to 4 min during 30 mW/cm^2^ ACXL (and thereby increasing the total energy dose from 5.4 to 7.2 J/cm^2^), significantly enhances corneal enzymatic resistance.^57^

Other treatment modifications have focussed on enhancing oxygen availability during ACXL through the use of an oxygen delivery device to boost the atmospheric oxygen concentration at the corneal surface,^65^ or a pulsed UVA exposure (cycling the UVA light on and off), which theoretically aids the replenishment of stromal oxygen concentrations when using higher irradiances.^66^ Indeed, it has been shown that the enzymatic resistance of porcine corneas cross-linked with 30 mW/cm^2^ UVA in a pulsed mode of 10-s on and 10-s off for a period of 8-min (total energy dose of 7.2 J/cm^2^) is slightly superior to that achieved when the same UVA intensity and energy dose is delivered in a non-pulsed manner.^57^ Many studies have reported that pulsed ACXL results in a significantly deeper stromal demarcation line than continuous ACXL,^67–70^ and that exposure to pulsed UVA can result in greater apoptotic effects and higher tissue damage than continuous delivery.^71,72^

In a study by Said *et al.* involving 49 progressive keratoconus patients, the use of pulsed ACXL [8-min exposure to 30 mW/cm^2^ UVA (1 s on/1 s off)] resulted in severe localised corneal haze and residual scarring in 19% patients.^70^ However, despite their scarcity, the majority of clinical trials involving pulsed ACXL are supportive of its use, showing a similar or increased efficacy compared with continuous ACXL with a follow-up time of up to 2 years.^67,73–75^ Most recently, in a large study involving a 2-year follow up of 870 patients (1192 eyes) cross-linked using pulsed ACXL [8-min exposure (1.5 s on/1.5 s off) to 30 mW/cm^2^ UVA], the treatment was shown to successfully halt keratoconus progression, with keratometric stabilisation being reported in 98.3% of eyes.^76^ All the aforementioned procedures have involved delivery of a broad beam of irradiation to the surface of the cornea. However, early results suggest that greater efficacy may be achieved using topography-guided customized cross-linking (PiXL), in which high fluence cross-linking is performed in a customizable pattern with the intensity based on the patients’ refractive error and corneal topography.^77^

## Modifications to the riboflavin solution

### Dextran-free riboflavin solutions

**Figure 2.**
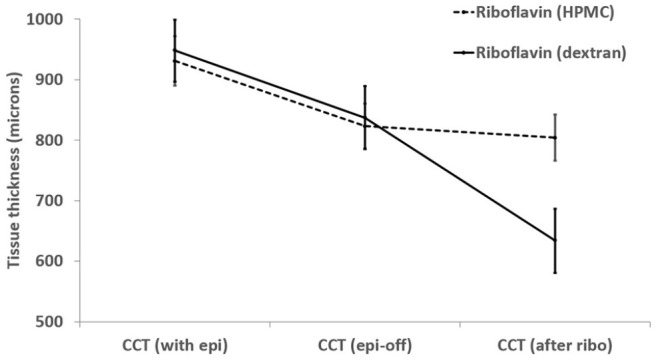
CCT of *ex vivo* porcine eyes (*n* = 40) measured with their epithelium intact (CCT with epi), after epithelium removal (CCT epi-off) and again, after a 20-min application of a 0.1% riboflavin solution in a carrier solution of 20% dextran or 1.1% HPMC (CCT after ribo). CCT, Central corneal thickness; HPMC, hydroxypropyl methylcellulose.

It has been shown, both experimentally and clinically, that the application of riboflavin solutions containing 20% dextran can lead to significant corneal thinning due to the deturgescent effect of the dextran.^56,78^ Although the reduction in corneal thickness may be rectified prior to UVA exposure by the application of a hypo-osmolar riboflavin solution, the use of dextran-free riboflavin solutions, in which hydroxypropyl methylcellulose (HPMC) is used as a replacement riboflavin carrier solution, are becoming increasingly popular alternatives. Unlike dextran, which has a high affinity for water and causes the cornea to dehydrate and thin, HPMC is a water soluble, viscoelastic polymer that has little effect on corneal hydration and thickness. Also riboflavin solutions containing HPMC have a faster diffusion rate and tend to require a much shorter soak time than those containing dextran.^79^ Our experimental findings from porcine eyes have shown that a small but nevertheless significant (*p* < 0.01) amount of corneal thinning occurs following a 20-min application of an isotonic riboflavin solution containing 1.1% HPMC but the thinning effect is much less than occurs with application of a riboflavin-dextran solution (Figure 2).

Clinically, the application of riboflavin-HPMC appears to have little effect on corneal thickness, causing it to decrease only slightly or, in some cases, increase.^80–82^ It has been suggested that a shallower depth of cross-linking may be expected with use of riboflavin-HPMC compared with riboflavin-dextran due to the differences in corneal thickness at the time of UVA exposure and the UVA transmission being higher in the thinner, riboflavin-dextran treated corneas.^79^ However, in a prospective RCT involving 40 eyes and a 2-year follow up, improvements in visual acuity and maximum keratometry were found following both standard cross-linking and ACXL (9 mW/cm^2^ UVA for 10-min) with riboflavin-HPMC.^83^ Similarly, in another RCT involving 48 eyes cross-linked with 3 mW/cm^2^ UVA for 30 min, no significant differences in maximum keratometry, visual acuity, corneal thickness or endothelial cell count were evident at 12-month follow up between eyes treated with riboflavin-dextran and those treated with riboflavin-HPMC.^81^ Although clinical studies are generally supportive of its use, further research is required into the long-term efficacy of riboflavin-HPMC/UVA cross-linking.

### Transepithelial riboflavin solutions

Transepithelial cross-linking, in which riboflavin is applied through an intact epithelium, has many potential benefits over the standard, epithelium-off procedure, being less invasive, carrying a reduced risk of infection and enabling easier treatment of paediatric and uncooperative patients. However, the effectiveness of the procedure is dependent upon there being a sufficient and homogenous distribution of riboflavin within the corneal stroma, a relatively unblocked transmission of UVA radiation through the epithelium and adequate stromal oxygen re-diffusion.

Laboratory studies have shown that minimal stromal penetration of riboflavin (containing dextran) occurs when the porcine epithelium is intact or even partially disrupted,^84–86^ as the high molecular weight of dextran inhibits the penetration of riboflavin across the epithelium. As a result, commercially available transepithelial riboflavin solutions tend to be dextran-free, with HPMC as a replacement carrier solution, and also contain permeation enhancers, such as benzalkonium chloride (BAC), trometamol or edetate disodium (EDTA), to loosen the tight junctions of the epithelial cells and facilitate passage of riboflavin into the stroma (Table 2). However, these additives can be toxic to the epithelium, with the impact being both duration and concentration dependent.^87^

**Table 2.** Commercially available CE-marked riboflavin solutions for keratoconus cross-linking.

	Riboflavin formulation	Composition
Epi-off, isotonic	Mediocross D	0.1% riboflavin 5-phosphate, 20% dextran
	Ricrolin	0.1% riboflavin 5-phosphate, 20% dextran
	Ribocross	0.1% riboflavin, 20% dextran
	Vibex Rapid	0.1% riboflavin-5-phosphate, HPMC
	Mediocross M	0.25% riboflavin-5-phosphate, HPMC
Epi-off, hypotonic	Mediocross H	0.1% riboflavin-5-phosphate
	Ricrolin+	0.1% riboflavin, EDTA, trometamol
Epi-on (Transepithelial)	Paracel	0.25% riboflavin-5-phosphate, HPMC, BAC, EDTA
	Mediocross TE	0.25% riboflavin-5-phosphate, HPMC, BAC
	Ricrolin TE	0.1% riboflavin-5-phosphate, dextran, trometamol, EDTA
	Ribocross TE	0.1% riboflavin-5-phosphate, dextran, Vitamin E-TPGS
	Ricrolin+	0.1% riboflavin-5-phosphate, trometamol, EDTA
	Ribofast	0.1% riboflavin-5-phosphate, Vitamin E-TPGS

It should be noted that the recommended application procedure varies greatly between different riboflavin solutions; e.g. 1 drop every 30 s for 15 min for Ribofast and Ribocross TE, 1 drop every 2 min for 30 min for Ricrolin TE and Ricrolin+, with the recommended soak time being reduced to just 5 min for an iontophoresis-assisted delivery of Ricrolin+.

BAC, benzalkonium chloride; EDTA, edetate disodium; HPMC, hydroxyl propyl methylcellulose; Vitamin E-TPGS, d-alpha-tocopheryl polyethylene-glycol succinate.

Although numerous laboratory studies have indicated that the cross-linking effect is greatly reduced in porcine corneas when transepithelial solutions are used compared with when riboflavin is applied directly to the stromal surface, the amount of cross-linking achieved may nevertheless be sufficient to stop keratoconus progression.^88,89^ Clinical studies of transepithelial effectiveness are notoriously difficult to compare due to differences in multiple aspects of the cross-linking procedure (riboflavin solution, UVA intensity and duration, etc.), as well as differences in the reported outcome measures and follow-up times. For example, Filipello *et al.* treated 20 patients using a transepithelial application of a riboflavin solution containing dextran, trometamol and EDTA, and an exposure to 3 mW/cm^2^ UVA for 30 min and reported that the treatment appeared to be safe and well tolerated, halted keratoconus progression and improved both visual and topographic parameters at 18-month follow up.^90^ Caporossi *et al.* performed the same procedure on 26 keratoconus eyes and reported improvements in visual acuity within the first 6-months post-treatment but a gradual return to pre-treatment levels and evidence of keratoconus instability and function regression within 24 months.^91^ In a prospective, interventional multi-centre cohort study involving 26 patients cross-linked transepithelially with a high concentration riboflavin solution containing BAC 0.01%, Gatzioufas *et al.* reported significant epithelium damage in the immediate post-operative period and evidence of keratoconus progression in 46% of eyes at 12-month follow up.^92^ Following a meta-analysis of 1-year follow-up outcomes from seven RCTs (total of 505 eyes) it was concluded that the standard procedure had a greater impact on halting keratoconus progression than transepithelial cross-linking.^93^ This finding has been supported by a 5-year follow-up study of 78 paediatric, progressive keratoconus patients in which it was shown that both standard cross-linking and transepithelial ACXL (using a 0.25% riboflavin solution containing HPMC and 0.007% BAC, and a 5-min exposure to 18 mW/cm^2^ UVA) successfully halted keratoconus progression, but the standard protocol was deemed safer and more effective.^94^

Recently, promising results have been reported by Zaheryani *et al.* in a double-blinded, randomised study in which 30 patients treated with epithelium-off ACXL (with a preservative and dextran-free riboflavin solution and a 10-min exposure to 9 mW/cm^2^ UVA) in one eye and, in the other, a Daya epithelium disruptor (Duckworth and Kent Ltd, Baldock, UK) was used to create tiny pores in the epithelium to facilitate the absorption of the same riboflavin solution into the stroma prior to cross-linking.^95^ The results at 12-month follow up showed that the potential for halting keratoconus progression did not differ between the two techniques.

**Figure 3.**
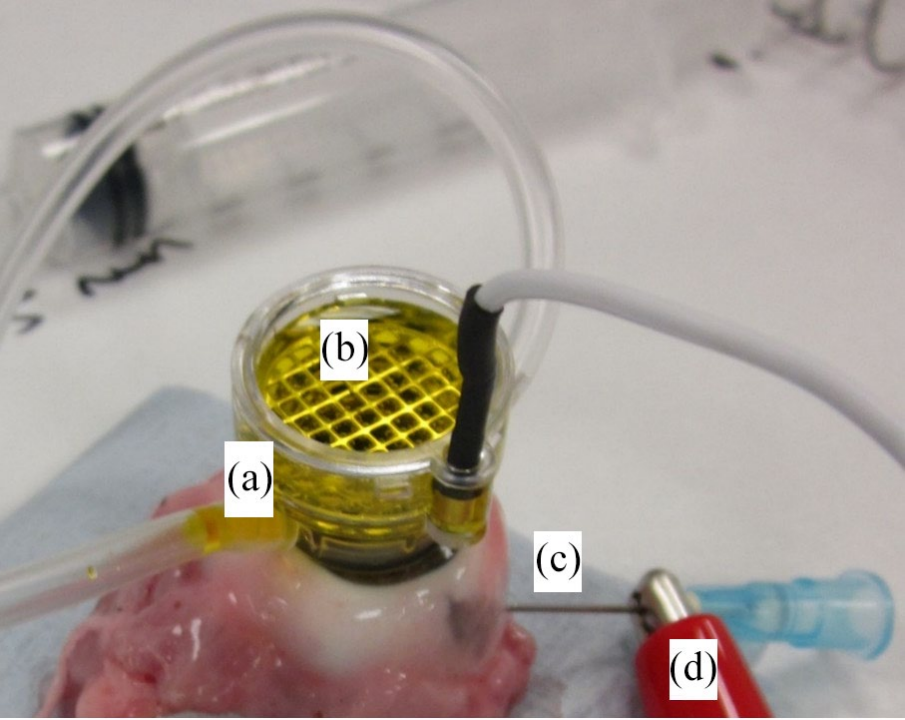
(a–d) Corneal iontophoresis involves the creation of a low intensity electric field to help transport negatively charged riboflavin solution across the intact epithelium. The corneal applicator is attached to the surface of the cornea by a vacuum suction system (a). The negative electrode is a steel grid (housed within the applicator) that is fully submerged within a reservoir of riboflavin (b). In our *ex vivo* system, a steel needle is inserted into the anterior chamber (c) and connected to the positive electrode (d), which returns to the power supply to complete the circuit. In the clinical situation, the positive electrode is attached to the patient’s forehead by means of a patch.

Iontophoresis-assisted riboflavin delivery represents another development in transepithelial cross-linking.^96^ With this technique, a low intensity electric field (0.5–1.0 mA) is used to facilitate the transport of the low molecular weight, negatively charged riboflavin solution across the intact epithelium and into the stroma (Figure 3). It has been demonstrated in rabbits that iontophoresis-assisted delivery of 0.1% riboflavin using a current of 1 mA for 5 min followed by a 30-min, 3 mW/cm^2^ UVA exposure, results in a stromal riboflavin concentration that is two-fold lower than that achieved with the standard, epithelium-off protocol but produces a similar increase in corneal stiffness and resistance to collagenase digestion.^97^ However, it should be noted that studies involving rabbit eyes provide a somewhat liberal estimate of transepithelial cross-linking efficacy because their corneal epithelium is thinner than that of the human cornea (~40 μm *versus* 50 μm), and studies using porcine eyes result in a more conservative measure due to their thicker epithelium (~90 μm). Indeed, contrary to the findings in rabbit eyes, it has been shown in porcine corneas that ACXL involving a 5-min iontophoresis-assisted delivery of riboflavin and 9 mW/cm^2^ UVA for 10 min does not produce the same level of enhanced resistance to enzymatic digestion as achieved with epithelium-off ACXL.^89^

Clinically, it has been shown that iontophoresis-assisted cross-linking produces significant visual improvements in the short term, but the corneal apex flattening is less than that achieved with epithelium-off approaches.^98,99^ Further to this, a 3-year follow up of paediatric patients found that the epithelium-off approach (using 10 mW/cm^2^ for 9 min) resulted in a cessation of keratoconus progression in 75% of eyes, whereas iontophoresis-assisted cross-linking slowed down keratoconus progression in only 50% of eyes.^100^

Based on studies of enzymatic resistance in cross-linked porcine corneas, it has been shown that the outcome of transepithelial cross-linking may be improved significantly through the use of higher riboflavin concentrations, a longer duration of iontophoresis and an increase in UVA radiance.^89,101^ These findings led to the development and optimisation of the St. Thomas’/Cardiff iontophoresis protocol,^57,88,89^ which essentially involves two transepithelial, iontophoresis-assisted deliveries of a 0.25% riboflavin solution with permeation enhancers (Mediocross TE Veni Vidi (Halifax, UK)), separated by a soakage period to allow time for stromal riboflavin diffusion, and followed by an exposure to 9 mW/cm^2^ UVA for 12 min and 30 s (total radiant exposure **6.75 J/cm**^2^). *In vitro*, this technique was found to be superior to that of other transepithelial protocols in terms of increasing resistance of the porcine cornea to enzymatic digestion and was closest to that of epithelium-off cross-linking.^89^ Further, long-term, randomised controlled studies are warranted to ascertain the true effectiveness of this, and other, transepithelial cross-linking procedures.

## Treatment modifications for very thin corneas

In advanced cases of keratoconus, the thickness of the cornea may be less than the 400 µm required for the standard cross-linking protocol. Various treatment modifications have been proposed for cross-linking such thin corneas, and these are covered comprehensively in a separate review.^102^ However, by way of a summary, they include the use of a hypo-osmolar riboflavin solution to swell the cornea prior to cross-linking^103,104^; the use of transepithelial riboflavin solutions and/or iontophoresis-assisted riboflavin delivery to maximise the thickness of the tissue^99,105,106^; the use of higher riboflavin concentrations to reduce the amount of UVA radiation reaching the deeper layers of the cornea and reduce the risk of endothelial toxicity^101^; the use of pachymetry-guided epithelial debridement (‘epithelial island technique’) to selectively remove the epithelium from regions of the cornea with a thickness >400 µm^107–109^; the use of a shielding riboflavin-soaked contact lens (‘contact lens-assisted cross-linking’)^110–112^; or the use of refractive stromal lenticules spread over the cornea to thicken the thinnest parts of the cornea (‘lenticule assisted cross-linking’).^113,114^ In addition to this, the potential of other non-UVA cross-linking procedures are being investigated. In particular, near infra-red illumination of a water-soluble bacteriochlorophyll derivative has been shown to produce a comparable enhancement of enzymatic resistance and tissue stiffness with that achieved with riboflavin/UVA cross-linking,^115–117^ and may in future offer a means of safely treating keratoconus corneas of any thickness.^118^ However, as is the case with all deviations from the standard cross-linking protocol, long-term studies and further research are required to validate the safety and efficacy of these treatments.

## Combination cross-linking ‘plus’ procedures for keratoconus

In a bid to effectively halt keratoconus progression and improve functional visual outcomes, the use of corneal cross-linking in combination with refractive procedures, such as topography-guided photorefractive keratectomy (PRK) and intra-corneal ring segment or continuous ring implantation, have become increasingly popular.^119–121^ Studies involving these combination therapies, collectively and commonly referred to as ‘CXL Plus’ procedures, have all shown some degrees of success.^122^ However, in many cases, the reliability of the evidence in these studies is limited by small sample sizes, the observational nature of the study design and the absence of control groups to demonstrate significant benefit of the combined procedure over and above that of the individual procedures. RCTs are therefore required to further confirm the long-term safety and effectiveness of these promising CXL Plus therapeutic approaches.
